# Disruption of Vps4 and JNK Function in Drosophila Causes Tumour Growth

**DOI:** 10.1371/journal.pone.0004354

**Published:** 2009-02-04

**Authors:** Lina M. Rodahl, Kaisa Haglund, Catherine Sem-Jacobsen, Franz Wendler, Jean-Paul Vincent, Karine Lindmo, Tor Erik Rusten, Harald Stenmark

**Affiliations:** 1 Centre for Cancer Biomedicine, Faculty of Medicine, University of Oslo and Institute for Cancer Research, the Norwegian Radium Hospital, Rikshospitalet University Hospital, Montebello, Oslo, Norway; 2 MRC National Institute of Medical Research, Mill Hill, London, United Kingdom; Ordway Research Institute, United States of America

## Abstract

Several regulators of endocytic trafficking have recently been identified as tumour suppressors in Drosophila. These include components of the endosomal sorting complex required for transport (ESCRT) machinery. Disruption of subunits of ESCRT-I and –II leads to cell-autonomous endosomal accumulation of ubiquitinated receptors, loss of apicobasal polarity and epithelial integrity, and increased cell death. Here we report that disruption of the ATPase dVps4, the most downstream component of the ESCRT machinery, causes the same array of cellular phenotypes. We find that loss of epithelial integrity and increased apoptosis, but not loss of cell polarity, require the activation of JNK signalling. Abrogation of JNK signalling prevents apoptosis in dVps4 deficient cells. Indeed double deficiency in dVps4 and JNK signalling leads to the formation of neoplastic tumours. We conclude that *dvps4* is a tumour suppressor in Drosophila and that JNK is central to the cell-autonomous phenotypes of ESCRT-deficient cells.

## Introduction

Correct receptor signalling and cell polarisation are required for proper organisation of epithelia. Signalling from ligand-bound receptors is modulated by endocytosis [1], [2], which mediates spatial restriction or downregulation of signalling [3]–[5] by sorting into intraluminal vesicles of multivesicular endosomes (MVEs) that fuse with lysosomes [6]–[9]. Both in the yeast Saccharomyces cerevisiae and in mammalian cells, Class E vacuolar protein sorting (Vps) proteins are important for sorting ubiquitinated receptors into the lumen of the MVEs. The class E *vps* yeast mutants accumulate vacuolar, endocytic and late-Golgi markers in an aberrant multilamellar endosome, the class E compartment [6], [9]–[13]. The majority of class E Vps proteins constitute the endosomal sorting complex required for transport (ESCRT) machinery, which is conserved in metazoans, including flies and mammals. ESCRT-I, -II and -III function cooperatively in endosomal receptor sorting and MVE biogenesis. Also important in this process is the ATPase Vps4, which functions to disassemble ESCRT-III multimers, thereby allowing the recycling of subunits [10], [14], [15]. The role of the ESCRT machinery in whole tissues has been particularly illuminated in the fruit fly Drosophila melanogaster in which disruption of ESCRT-I or -II causes cell-autonomous loss of polarity, cytoskeleton disruption and apoptosis. ESCRT-deficient cells also activate Notch signalling, which triggers the release of a cytokine thus activating proliferation in adjacent, normal cells [16]–[19]. In this paper, we focus on the cell autonomous effects caused by ESCRT deficiency.

Even though it is established that the ESCRTs control tissue organization, the mechanisms that mediate this activity remain unclear. Here we explore the effects of reducing the activity of Drosophila Vps4 (dVps4), the most downstream component of the ESCRT machinery. In the main, dVps4 activity was knocked down with a dominant negative construct carrying a point mutation in the catalytic site [14]. Supportive evidence was also obtained with a small genomic deletion and an RNAi hairpin construct. We find that several cell-autonomous effects observed when ESCRT function is disrupted are mediated by c-Jun N-terminal kinase (JNK), a member of the mitogen-activated protein kinase (MAPK) family, which has extensively been implicated in programmed cell death [20] and is misregulated in many types of mammalian cancers [21]–[25]. While reduction in either dVps4 activity or JNK signalling does not cause overgrowth, double deficiency leads to the formation of neoplastic tumours.

## Results

### CG6842 encodes the Drosophila orthologue of Vps4

**Figure 1:**
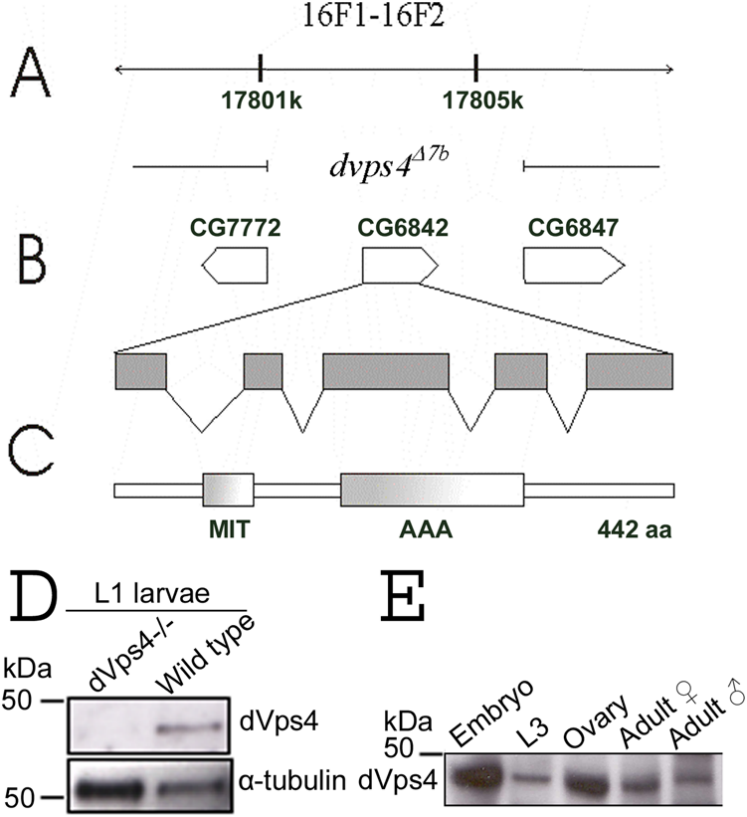
Characterisation of Drosophila Vps4 (dVps4). (A) Diagram of the genomic region surrounding *dvps4*/CG6842 located at band 16F1–16F2 around 17801–17805 kbp on the X-chromosome. (B) dVps4 coding sequence with close by genes. The region deleted indicates the genotype of the mutant *dvps4^Δ7b^*. dVps4/CG6842 is shown with exon-intron boundaries. (C) dVps4 protein (442 aa, 49,6 kD). The AAA-domain (ATP-binding) and the MIT-domain (recognises substrates such as ESCRT-III subunits) are conserved domains. (D) An antibody raised against dVps4 recognised a single protein of expected size in wild-type L1 larvae (lane 2) but not in dVps4 L1 mutant larvae (lane 1). Tubulin served as loading control. (E) dVps4 was detected in all stages of development (L3, third instar larvae).

The Drosophila genome contains a single Vps4 homologue encoded by the CG6842 gene, here referred to as *dvps4*, which contains four exon-intron boundaries and is located between two genes of unknown function on the X-chromosome (Fig. 1A,B). The predicted dVps4 protein contains 442 amino acid residues and displays 75.1% identity to human Vps4b, 73.7% identity to human Vps4a and 58% identity to yeast Vps4p (Supporting Fig. S1). There is no significant similarity to other proteins in the database. The dVps4 protein contains an AAA-ATPase domain and a Microtubule interacting and transport (MIT) domain (Fig. 1C) [14], [15]. For our initial studies of the function of dVps4 we constructed a small deficiency allele, *dvps4^Δ7b^*. In this allele, the whole coding region and flanking sequences were deleted by FRT-mediated recombination between two FRT-containing P-elements, *f07379* and *d09806* carried in trans (Fig. 1A, see Materials and Methods) [26]). An antibody raised against dVps4 recognised a protein with the predicted size on Western blots from lysates of wild type L1 control larvae, but not from L1 *dvps4^Δ7b^* larvae, indicating that the antibody is specific and that dVps4 protein is indeed absent in the mutants (Fig. 1D). In the wild type, dVps4 protein was found to be present at all stages of development, from embryos to adult males and females (Fig. 1E), suggesting a pleiotropic role. We found that *dvps4^Δ7b^* homozygous animals died at the first larval instar similar to *tsg101*/*erupted* (*ept*) and *vps25* homozygous mutants [19], [27]. This is likely because *dvps4* is an essential gene although an essential contribution from the two neighbouring genes cannot be excluded (Fig. 1B). All attempts to rescue the *dvps4^Δ7b^* mutants by expression of a dVps4 wild type construct failed. This was probably due to the dominant negative effect that is caused by overexpression of this construct (see below).

### dVps4 disruption causes accumulation of ubiquitinated proteins and disruption of epithelial organisation

**Figure 2:**
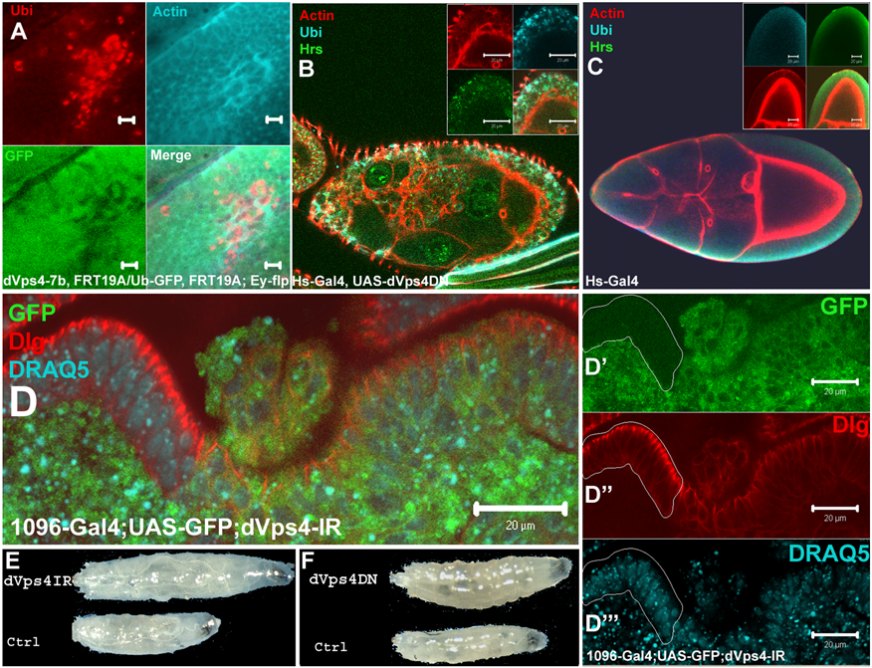
dVps4 disruption leads to accumulation of ubiquitinated cargo and loss of cell polarity. (A) A dVps4 eye disc mutant clone lacking GFP. Ubiquitin is accumulated (red) and actin was dysregulated (blue). (B) An overview of a Drosophila egg chamber and close up (inset in B). Expression of UAS-dVps4-DN under a Hs-Gal4 driver similarly to the *dvps4^Δ7b^* clone led to accumulation of ubiquitin (blue) and Hrs (green) in enlarged endosomal structures in addition to dysregulation of actin. (C) A Hs-Gal4 control egg chamber stained for F-actin (red), Ubiquitin (blue) and Hrs (green). (D) Cells expressing dVps4-IR; CD8-GFP lost the cell polarity marker Dlg (red) staining (D and split image D′–D′′′). The wild type cells lacking GFP are outlined in D′–D′′′. Nuclei are stained with DRAQ5 in blue (D′′′). Scale bars 20 µm. (E and F) The larvae expressing either dVps4-IR or dVps4-DN induced by the 1096-Gal4 driver grew to a much larger size than the dVps4-IR and -DN controls, lacking the driver.

Patches of dVps4-deficient cells (homozygous *dvps4^Δ7b^*) were generated in developing eye imaginal discs. These cells showed an apparent loss of epithelial organisation and accumulated ubiquitin in punctuate structures (Fig. 2A), phenotypes that are also seen as a result of disruption of the ESCRT-I component *ept/tsg101*, or of the ESCRT-II component *vps25* [16], [18], [19], [27]. dVps4 mutant cells also displayed a dramatic dysorganization of the actin cytoskeleton similar to that reported in *dvps28* (ESCRT-I) mutant cells [17]. Mostly small (1–2 cells) *dvps4^Δ7b^* mutant clones could be recovered, probably because the FRT-mediated recombination events used to create mutant patches is triggered in individual cells and that, in isolation, *dvps4^Δ7b^* mutant cells undergo apoptosis faster than they can grow. In order to assess the contribution of dVps4 to the phenotype of *dvps4^Δ7b^* homozygous cells, we constructed a Gal4-dependent transgene to express wild type dVps4 that could be used in a rescue experiment. Expression of this transgene in wing imaginal discs caused accumulation of endocytic cargo, polarity disruption and cell death (data not shown). These are characteristics of the loss of function phenotype and it is therefore likely that overexpressed wild type protein has a dominant negative effect on ESCRT activity. Since the *dvps4^Δ7b^* mutant clones are so short lived and because of the possibility that the flanking genes might be affected by the deletion, we turned to RNAi-mediated knock-down [28] and a dominant negative form of dVps4, dVps4-DN as means of reducing dVps4 activity. Since both the RNAi and the DN constructs can be expressed under Gal4 control and since these constructs are likely to create a hypomorphic situation, large patches of deficient tissue can be generated thus overcoming the problem of growth from a single mutant cell. As a first step, heat-shock-inducible Gal4 was used to activate the UAS-dVps4-DN transgene ubiquitously, and egg chamber phenotypes were scored. As can be seen in Fig. 2B, this caused the accumulation of the endosome marker Hrs and conjugated ubiquitin on large structures within expressing cells. In addition we used phalloidin staining to visualize F-actin and found that the cytoskeleton was disrupted with tangles of abnormal accumulation. The same phenotype was seen in *dvps4^Δ7b^* patches within the follicle epithelium and was also seen in wing imaginal disc cells expressing dVps4-IR driven by 1096-Gal4, a wing pouch driver (not shown). This phenotype was not observed in egg chambers only expressing the Hs-Gal4 driver upon heat shock (Fig. 2C). We conclude that, since dVps4-DN and dVps4-IR affect the endosomal distribution of ubiquitinated proteins, these constructs are likely to interfere with ESCRT activity as intended.

Having established that a reduction in dVps4 activity causes trafficking defects expected from ESCRT deficiency, we set out to ask if it also causes disruption of cell polarity and loss of epithelial integrity [16]–[19] as is seen following loss of ESCRT-I and -II components. Wing discs expressing dVps4-IR were stained with an antibody against Discs-large (Dlg) which labels the lateral membrane domains. Dlg was reduced in dVps4-IR expressing cells as compared to the situation in surrounding wild type tissue (Fig. 2D–D′′′, where dVps4-IR-expressing cells are marked by GFP expression). This phenotype suggests loss of cell polarity and epithelial integrity. Larvae expressing dVps4-IR or dVps4-DN in the wing disc continued to grow beyond the normal time of growth termination, became oversized and died shortly after delayed pupation (Fig. 2E,F).

**Figure 3:**
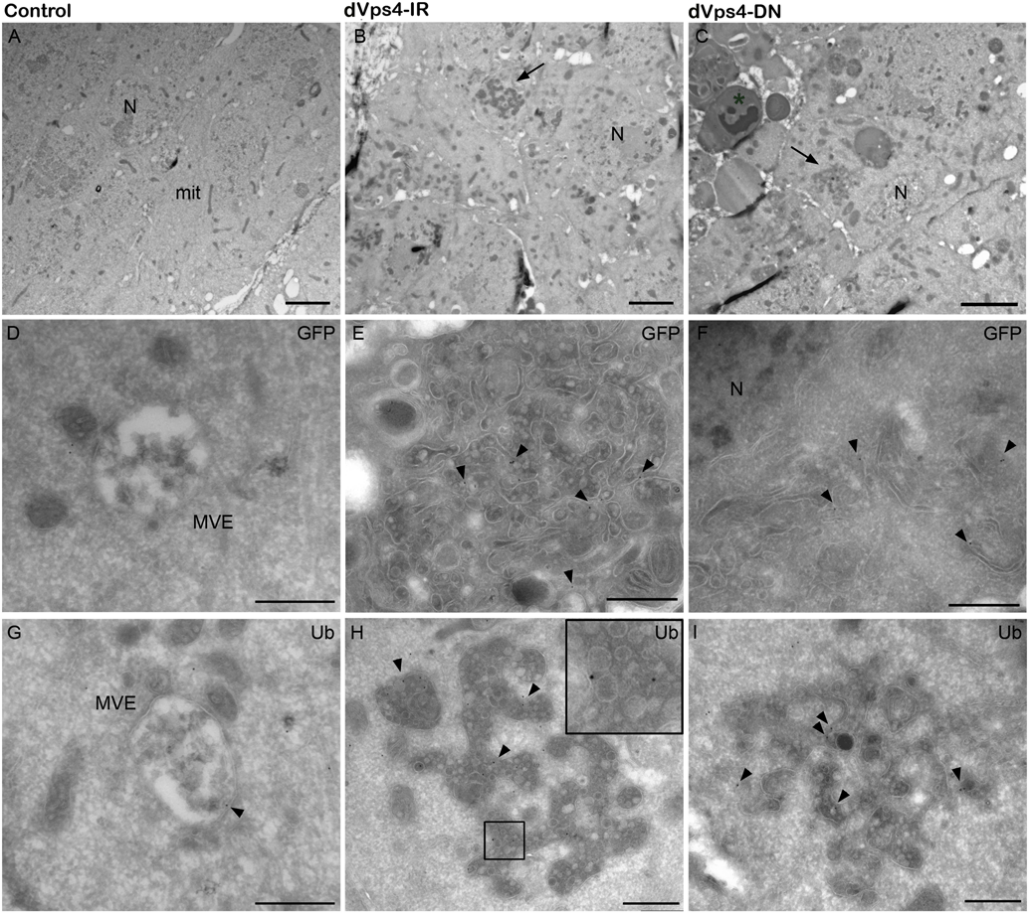
Ultrastructural characterisation of dVps4-DN and dVps4-IR-expressing cells shows enlarged MVEs containing accumulated ubiquitinated cargo. (A) Overview of control wing discs 1096-Gal4; CD8-GFP that contain normal MVEs as shown in the close-ups (D and G). The MVEs did not accumulate the CD8-GFP marker (D), but some ubiquitin labeling was present (arrow in G). (B) In the 1096-Gal4; CD8-GFP; dVps4-IR and (C) dVps4-DN discs large dense structures were observed (arrows) containing intraluminal vesicles, often with irregular shape. These structures sometimes occupied most of the cell volume. In both cases GFP accumulation was observed (arrows in E and F) and also strong labeling against ubiquitin (arrows in H and I). In the 1096-Gal4; CD8-GFP; dVps4-DN discs an increased number of apoptotic cells were observed (asterisks in C), in contrast to the dVps4-IR discs, where less apoptotic cells were present (not shown). Scale bars; A–C, 3 µm; D–I, 500 nm.

To investigate the ultrastructural defects of dVps4-deficient cells, we performed immuno-electron microscopy (EM) on wing imaginal discs expressing dVps4-DN or dVps4-IR. As a membrane marker we used CD8-GFP (detected with gold-labeled anti-GFP) expressed from a transgene. This transgene also allowed cells expressing the knockdown construct to be identified unambiguously within the tissue. Expression of either dVps4-IR or dVps4-DN caused the formation of enlarged tubular structures, which were not seen in control discs (Fig. 3A,B,C). Both CD8-GFP and ubiquitin accumulated on these structures, suggesting that they represent enlarged late endosomes. Interestingly, the Notch receptor also accumulated on these structures (data not shown), which could therefore be a site of Notch signalling. As could be seen upon closer examination, in addition to accumulating ubiquitin and being positive for CD8-GFP, these enlarged MVE-like structures contained internal vesicles (Fig. 3E,F,H,I). Perhaps residual dVps4 activity allows low-level endovesiculation to proceed. As in *dvps4^Δ7b^* mutant tissue, an increased number of apoptotic cells were seen in the domain of dVps4-DN expression (asterisk, Fig. 3C). Apoptosis was also enhanced in response to dVps4-IR expression (data not shown), however this was less pronounced than with dVps4-DN. This might be because the DN construct inhibits dVps4 activity more thoroughly than dVps4-IR.

Larval overgrowth, epithelial disorganisation, and increased apoptosis are characteristic of mutants in tumour suppressor genes such as *scribble* (*scrib*), *dlg*, *lethal giant larvae* (*lgl*) [29] *avalanche* (*avl*) [30] and three other components of the ESCRT machinery, *ept*/*tsg101* [27], *vps25* [16], [18], [19] and *vps28* [17]. Therefore, dVps4 bears the hallmarks of a tumour suppressor.

### dVps4 disruption causes upregulation of JNK, integrin and MMP1

**Figure 4:**
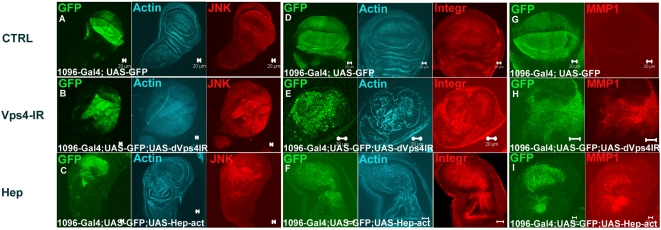
Disruption of dVps4 function leads to increased JNK signalling. (A, D and G) Wing disc control (A) stained for F-actin (blue) and pJNK (red), (D) F-actin (blue) and Integrin (red) and (G) MMP1 (red). (B, E and H) dVps4-IR (B and E) showed a disrupted F-actin (Blue) phenotype, (B) upregulated pJNK (red), (E) Integrin disruption (red) and (H) upregulated MMP1 levels (red). The phenotype phenocopied the effect given when Hemipterous (JNKK) was expressed (C) stained for F-actin (Blue) and pJNK (red), (F) F-actin (blue) and Integrin (red) and (I) MMP1 (red). Scale bars 20 µm.

As mentioned above, apoptosis is increased in cells expressing dVps4-DN or dVps4–IR, as well as in *tsg101* and *vps25* mutant cells [16], [18], [19], [27]. Little is known so far about the signalling pathway that mediates this effect. One candidate pathway is that controlled by JNK since it is known to activate apoptosis in a variety of situations ([31] and references therein). Indeed, in dVps4-IR-expressing cells, the levels of phosphorylated active JNK (pJNK) were found to be upregulated in comparison to control discs (Fig. 4A,B). Consistent with this observation, JNK downstream targets such as βPS integrin (myospheroid) and matrix metalloprotease 1 (MMP1) were ectopically expressed (Fig. 4E,H), a phenotype also seen in cells that express an activated form of Hemipterous, the Drosophila JNK kinase (Fig. 4C,F,I). Moreover, expression of dVps4–IR caused a similar disorganisation of the actin cytoskeleton as activated JNK (Fig. 4B,C,E,F) [32]. None of the above proteins were seen to be upregulated in the control discs expressing only CD8-GFP (Fig. 4A,D,G). Overall therefore, it appears that JNK signalling is upregulated following expression of dVps4–IR (or dVps4–DN; see below).

**Figure 5:**
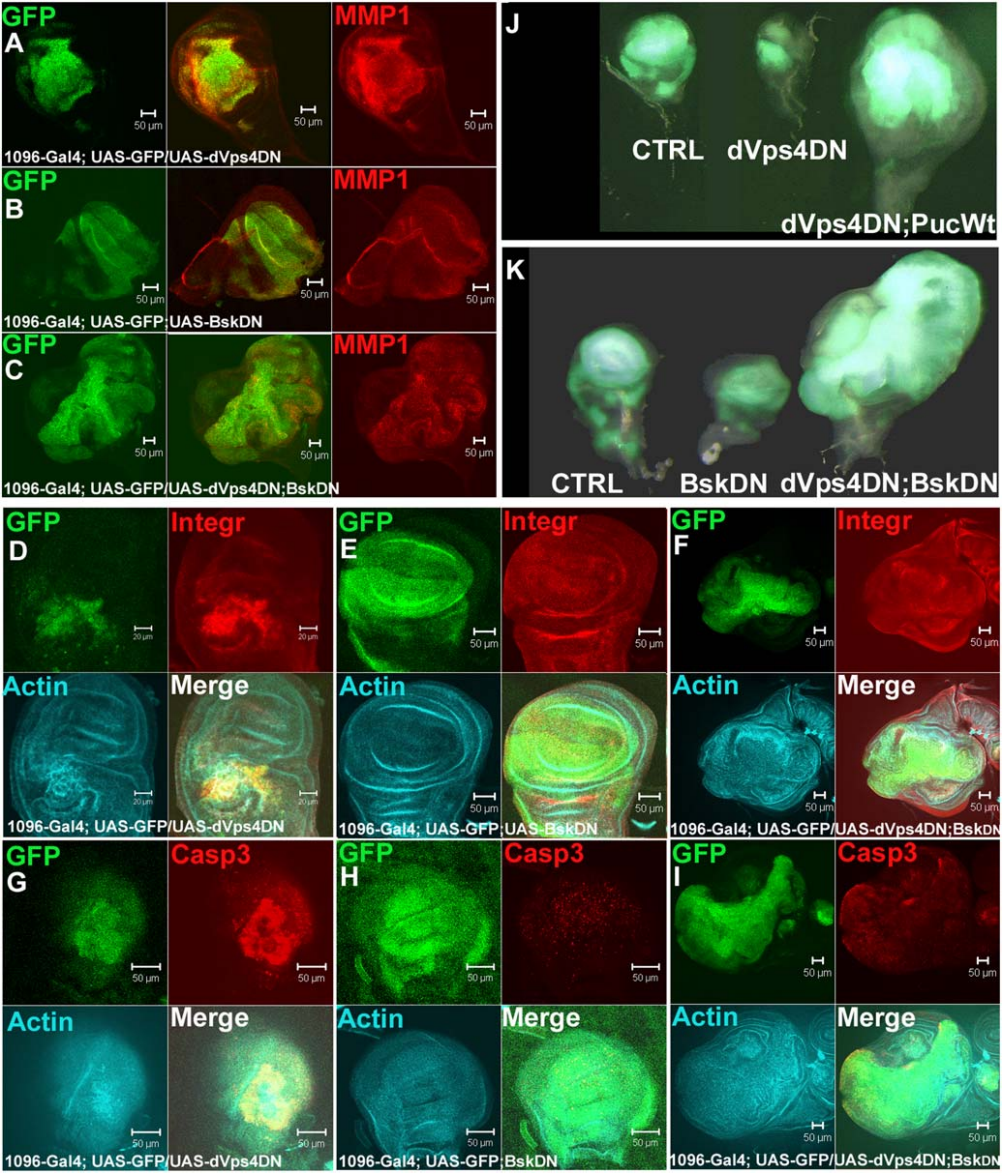
Downregulation of JNK signalling in dVps4-deficient cells leads to cell survival and tumour-like growth. (A) dVps4-DN expression in wing discs marked by GFP led to upregulation of MMP1 (red) and (D) β-integrin (red), (D and G) disruption of actin (blue) and (G) activation of Caspase-3 (red), compared to (B, E and H) expression of Bsk^DN^ alone. (C, F and I) When the JNK pathway was blocked by expression of Bsk-DN together with dVps4-DN, (C) MMP1, (F) β-integrin and (I) activated Caspase-3 were reduced. (F and I) The F-actin condensation was reduced. (J and K) The dVps4-DN; Bsk-DN and dVps4-DN; puc cells survived longer and the tissue displayed tumour-like growth compared to Bsk-DN and 1096; CD8-GFP expression alone. Scale bars are 50 µm in all pictures except D, scale bar 20 µm.

Since activation of JNK on its own produced similar effects to those caused by loss of dVps4, we reasoned that JNK signalling may be a key mediator of the defects observed in dVps4 deficient cells. To test this hypothesis we asked if the phenotypes caused by expression of dVps4-DN are suppressed by concomitant reduction of JNK signalling. Like dVps4–IR, dVps4-DN caused pronounced upregulation of MMP1 and integrin expression as well as a striking disruption of the actin cytoskeleton (Fig. 5A,D,G). Concomitant suppression of JNK signalling (by co-expression of a dominant negative from of JNK, *basket* ^DN^ (Bsk-DN) [33] brought back actin, MMP1 and integrin expression closer to wild type levels (Fig. 5C,F). Co-expression of the JNK phosphatase *puckered* (*puc*), a negative regulator of JNK signalling, had a similar, albeit weaker effect (data not shown). Actin, MMP1 and integrin were neither upregulated nor disrupted in discs expressing Bsk-DN alone (Fig. 5B,E). Therefore upregulation of actin, MMP1 and integrin expression in dVps4-DN-expressing cells is likely mediated by JNK signalling.

Another important consequence of reduced dVps4 activity is an increase in apoptosis. This was noticeable in dVps4–IR-expressing discs (not shown) and was striking in tissues expressing dVps4–DN (Fig 5G). As expected, dVps4–DN-expressing discs were much reduced in size. Such size reduction required JNK signalling since co-expression of Bsk-DN restored growth in dVps4-DN-expressing discs. In fact, discs co-expressing dVps4–DN and Bsk-DN overgrew and reached a much larger size than control, wild type discs (Fig. 5J,K). A similar effect is seen in discs co-expressing dVps4–DN and puc. Overgrowth requires inhibition of both dVps4 and JNK signalling since discs expressing either Bsk-DN or puc alone are reduced in size (Fig. 5K, not shown). In order to test whether suppression of JNK signalling enables overgrowth of dVps4-deficient cells by suppressing apoptosis, anti-activated Caspase-3 stainings were performed. Only sporadic low levels of activated Caspase-3 were observed in discs expressing Bsk-DN alone (Fig. 5H). Importantly, high level staining detected in discs expressing dVps4-DN alone (Fig. 5G) was strongly suppressed by coexpression of Bsk-DN (Fig. 5I). We conclude that increased apoptosis caused by loss of dVps4 function is mediated by activation of JNK signalling. However, not all phenotypes caused by dVps4 deficiency require JNK signalling. As shown in Supporting Fig. S2, the downregulation of Dlg seen following expression of dVps4-DN is not suppressed by coexpression of Bsk-DN.

## Discussion

In this study we have shown that reduced activity of the ESCRT-III regulator dVps4 causes the same array of cellular phenotypes as those seen in cells lacking ESCRT-I or -II components. These phenotypes include upregulation of MMP1 and integrin, dysregulation of the actin cytoskeleton, increased apoptosis, and loss of apico-basal polarity. In addition, we found that dVps4 knockdown also leads to activation of JNK signalling. In fact, JNK signalling appears to be a central mediator of many of the cell-autonomous phenotypes observed in dVps4-deficient cells. Indeed, all the above phenotypes, except for the loss of apico-basal polarity, are alleviated by concomitant suppression of JNK signalling.

Importantly, apoptosis in dVps4-expressing tissue was strongly counteracted by downregulation of JNK signalling (Fig. 5). Overexpression of Bsk-DN, or of the JNK phosphatase puc, led to a reduction of Caspase-3 staining and increased cell survival in tissue expressing dVps4-DN. By contrast, blocking JNK signalling by expressing puc did not prevent apoptosis in *vps25* null mutant cells [16]. The difference could be due to the extent of loss of ESCRT function in the two situations since dVps4-DN is likely to cause a hypomorphic situation. Our results thus show that JNK is central to the induction of apoptosis under conditions of partial loss of ESCRT activity. Strikingly, simultaneous reduction of dVps4 and JNK signalling leads to the formation of neoplastic tumours. *dVps4* can therefore be considered a tumour suppressor gene. Importantly, such tumour suppressor activity is revealed only when activity is partially lost, perhaps because dVps4 has a pleiotropic role; complete loss of function would impede an essential cell biological activity and thus prevent growth while partial loss of function would unbridle a growth inhibiting mechanism.

Previous studies of *vps25* and *tsg101* Drosophila mutants have revealed a strong hyperplastic growth of tissue adjacent to the mutant cells [16], [18], [19], [27]. Such an effect is quite distinct from the cell autonomous overgrowth reported in this manuscript, which requires inhibition of JNK signalling. Non-autonomous overgrowth triggered by *vps25* and *tsg101* loss of function has been attributed to upregulated cytokine secretion, following autonomous activation of the Notch signalling pathway in the mutant cells [16], [18], [19]. We have also observed an activation of Notch signalling and cell non-autonomous hyperplastic growth in tissues flanking dVps4-IR-expressing cells (data not shown), suggesting that non-autonomous stimulation of proliferation may be a common feature of ESCRT deficiency. We suggest that both the non-cell-autonomous and the cell autonomous effects of dVps4 disruption contribute to tumourigenesis.

In conclusion, we have shown that the ESCRT-III regulator dVps4 is crucial for attenuation of signalling pathways that mediate cell-autonomous apoptosis, actin rearrangement, integrin upregulation, MMP1 upregulation and loss of polarity. With exception of the latter, all these effects can be attributed, entirely or partially, to activation of JNK. Further studies aimed at clarifying the mechanistic relationship between dVps4 inactivation and JNK activation will be required in order to fully understand the tumour suppressor function of dVps4.

## Materials and Methods

### Fly strains

w1118;pMR[ w+, UAS-vps4/CG6842]-IR-2 # 35126 (Vienna Drosophila RNAi Center). dVps4DN was constructed as described in [34]. *dvps4^Δ7b^* Frt19 mutant clones were generated by using yw, Ubi-GFP,w+, FRT19A; eyflp(II) (LO-GFP clones) from D. Bilder, w; P [GAL4-Hsp70] 2/CyO, w,p[1096-Gal4]; UAS-GFP (Bloomington stock centre #2077 and #8696 respectively), UAS-Hep-act, UAS-BskDN and UAS-Puc-wt were kindly given to us by Dirk Bohmann.

### Generation of *dvps4^Δ7b^* Drosophila mutants

The *dvps4^Δ7b^* allele was generated by using P-transposase-based deletion by FRT-site recombination between the two transposon insertions f07379 id: AY515148 (WH p-element) and d09806 id: AY515149 (XP p-element) situated on opposite sides of the *dvps4* gene locus [26] (a gift from Ruth Palmer) and Hs-FLP and P[w+]/FM7c, w−.

### Immunohistochemistry and microscopy

Antibodies against dVps4 were generated by immunising two rabbits with a mixture of the two peptides C-DLVTKATEEDRNKN and CSARSDNENDSVRRI, coupled to LPH. (BioGenes GmbH, Germany). Antibodies were affinity purified using the same two peptides coupled to Sulfo-Link (Pierce). Imaginal discs were fixed and stained according to standard protocols [34]. The following antibodies and their dilutions were used for immunohistochemisty: Mouse monoclonal antibodies against conjugated ubiquitin (FK2) (1∶1000) (Affinity BioReagents, UK.), guinea pig anti-Hrs (1∶1000) [35], mouse anti-Dlg 4F3 (1∶200) (DSHB), rabbit anti-active JNK (1∶1000) (Promega), mouse anti-Integrin #CF.6G11 (1∶200) (DSHB), mouse anti-MMP1 [DSHB 3A6B4/5H7B11/3B8D12; the three different antibodies were mixed in amounts (1∶1∶1)] (1∶25). Tissue samples were counterstained by DRAQ5 (1∶1000) (Alexis biochemicals) to visualize DNA and Alexa Fluor® 647 phalloidin (1∶40) or Rhodamine phalloidin (1∶400) (Invitrogen) to visualize actin. Tissues were examined using a Zeiss LSM 510 META confocal microscope. Images were prepared with Zeiss LSM Image Browser (Version 3.2) and Adobe Photoshop (Version 7.0).

### Western blot analysis

For Western blot, 25 *dvps4* mutant L1 larvae, 25 wild type L1 larvae or indicated Drosophila tissues were collected and homogenized in ice-cold lysis buffer (50 mM Tris pH 8, 150 mM NaCl, 0.5% NP-40) containing protease inhibitor cocktail (Complete, EDTA-free, Roche). Lysates were cleared by centrifugation at 4°C for 15 min at 13,000 rpm. For each sample 20 µg of protein (Quant-iT™ Protein Assay Kit, Invitrogen) was resolved by 10% SDS-PAGE, followed by Western blot. Affinity purified rabbit anti-Vps4 antibodies were used at 1∶500, mouse α-tubulin antibodies at 1∶10000 (Sigma) and secondary HRP-conjugated anti-rabbit and anti-mouse antibodies at 1∶5000 (Jackson ImmunoResearch).

### Electron microscopy

L3 larvae were fixed in 4% formaldehyde/0.1% gluteraldehyde in 0.1 M phosphate buffer at room temperature for 60 min, washed and the wing disc dissected out. This was embedded in 12% gelatin and infiltrated with 2.3 M sucrose, mounted on silver pins, and frozen in liquid nitrogen. Ultrathin cryosections were cut at −110°C (EM FCS ultramicrotome; Leica) and collected with a 1∶1 mixture of 2% methyl cellulose and 2.3 M sucrose. Sections were transferred to formvar/carbon-coated grids and labeled with antibodies against GFP, Notch or Ubiquitin, followed by Protein A conjugates essentially as described [36]. Sections were observed at 60–80 kV using a JEOL JEM-1230 electron microscope, equipped with a Morada camera.

## Supporting Information

Figure S1 Vps4 protein alignment. Vps4 protein sequence alignment of Drosophila melanogaster (NP_573258), Homo sapiens (NP_004860), Saccharomyces cerevisiae (NP_015499) and Arabidopsis thaliana (NP_180328)-Vps4 respectively.
(0.99 MB TIF)

Figure S2 Cell polarity is not restored by inhibition of JNK signalling in dVps4-DN cells. Dlg (red) staining is still weaker and polarity not restored when JNK-DN is coexpressed in dVps4-DN expressing cells (GFP positive).
(2.21 MB TIF)
