# Supplementary figures and images for: Disruption of Vps4 and JNK Function in Drosophila Causes Tumour Growth
Source: PLoS One. 2009 Feb 4;4(2):e4354. doi: 10.1371/journal.pone.0004354 (PMC2632753; doi:10.1371/journal.pone.0004354)

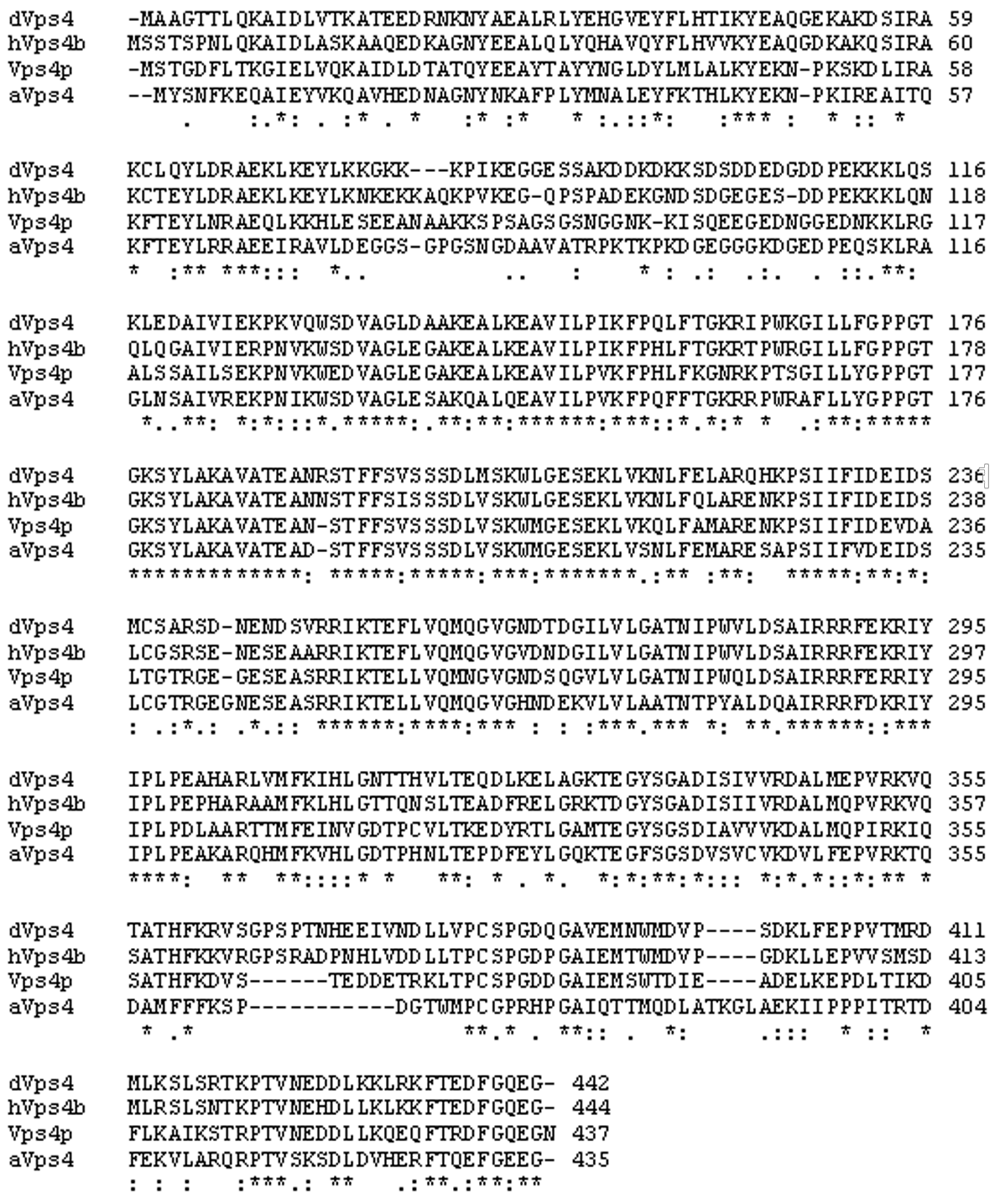

Supplement: Figure S1 — Vps4 protein alignment. Vps4 protein sequence alignment of Drosophila melanogaster (NP_573258), Homo sapiens (NP_004860), Saccharomyces cerevisiae (NP_015499) and Arabidopsis thaliana (NP_180328)-Vps4 respectively. (0.99 MB TIF) [file pone.0004354.s001.tif]

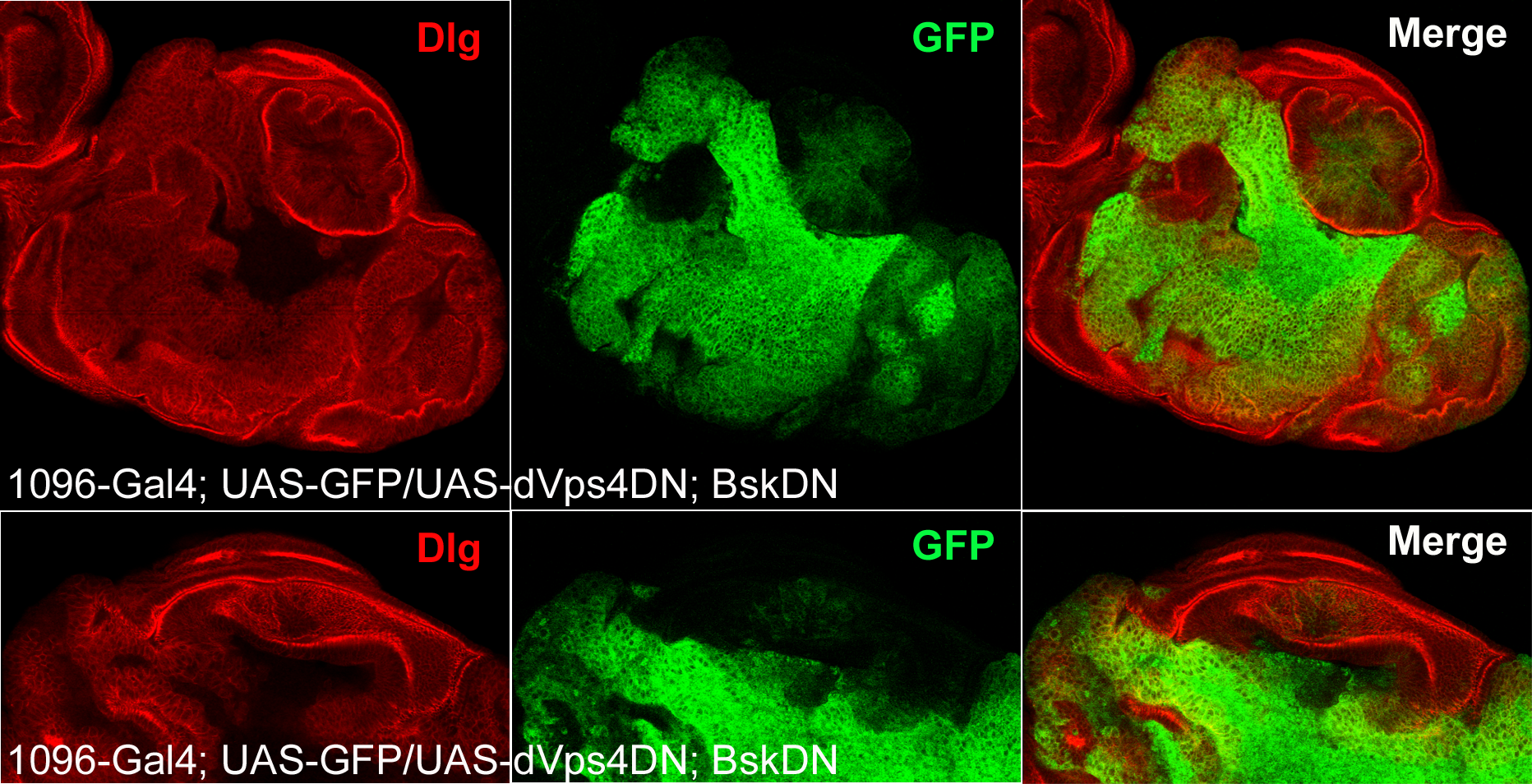

Supplement: Figure S2 — Cell polarity is not restored by inhibition of JNK signalling in dVps4-DN cells. Dlg (red) staining is still weaker and polarity not restored when JNK-DN is coexpressed in dVps4-DN expressing cells (GFP positive). (2.21 MB TIF) [file pone.0004354.s002.tif]
